# Supplementary material for: Biochemical Characterization of Orange-Colored Rice Calli Induced by Target Mutagenesis of OsOr Gene
Source: Plants (Basel). 2022 Dec 22;12(1):56. doi: 10.3390/plants12010056 (PMC9823629; doi:10.3390/plants12010056)
Supplement: Supplementary file 1 [file plants-12-00056-s001.zip › Supplementary Tables and Figures V1.docx]

**Supplementary Data**

**Biochemical characterization of orange-colored rice calli induced by target mutagenesis of *OsOr* gene**

Hee Kyoung Kim, Jin Young Kim, Jong Hee Kim, Ji Yun Go, Yoo-Seob Jung, Hyo Ju Lee, Mi-Jeong Ahn, Jihyeon Yu, Sangsu Bae, Ho Soo Kim, Sang Soo Kwak, Me-Sun Kim Yong-Gu Cho, Yu Jin Jung* and Kwon Kyoo Kang*

**Contents**

**Supplementary Table S1.** Primers for deep sequencing and qRT-PCR were used in this study.

**Supplementary Table S2.** Mutation frequencies at on-target and potential off-target sites among selected sgRNA targets of the *OsOr* gene in rice genome using CRISPR RGEN tools (http://www.rgenome.net/cas-designer/). PAM is presented in red, and the selected sgRNA is underlined.

**Supplementary Table S3**. Mutation percentage and types of targeted mutagenesis of *OsOr* gene using the CRISPR-Cas9 system.

**Supplementary Table S4.** Analysis of carotenoid content. Carotenoids were extracted from the WT, OC#1, OC#3, and OC#17 lines using a 0.01% solution of butylated hydroxytoluene in acetone. and analyzed using an Agilent 1260 high-performance liquid chromatography (HPLC) system.

**Supplementary Table S5.** Statistical analysis of carotenoid contents.

**Supplementary Table S6.** Statistical analysis of relative expression level of carotenoid pathway genes.

**Supplementary Table S7.** Statistical analysis of salt-mediated oxidative stress on transgenic calli lines.

**Supplementary Figure S1.** PCR analysis using bar-gene-specific primers to confirm the transgenic callus lines. M, molecular marker; PC, positive control; WT, wild-type; DW, water.

**Supplementary Figure S2.** Photographs of the subculture process for the generation of a single callus line. The OC#1, OC#3, and OC#17 lines were selected by targeted mutagenesis using the CRISPR-Cas9 system. These lines were subjected to several subcultures for a single cell line and maintained in AA medium.

**Supplementary Table S1.** Primers for deep sequencing and qRT-PCR were used in this study.

| Primer | Primer sequence (5’-3’) | Purpose |
| --- | --- | --- |
| *OsOr*_sg1 1st-Fw | CTGGCACATCTTCTTTCTGC | NGS analysis for *OsOr*_sg1 |
| *OsOr*_sg1 1st-Rv | CCTCCCACACCGAGTTTAAG |  |
| *OsOr*_sg1 2nd-Fw | CGTAGATTCGCAGGTTGAGA |  |
| *OsOr*_sg1 2nd-Rv | AATGAGCTGATTTGGAACTTCA |  |
| *OsOr*_sg2 1st-Fw | TATCCATCCTTCCTCCGTCA | NGS analysis for *OsOr*_sg2 |
| *OsOr*_sg2 1st-Rv | ATCCTCCATCAACAGCGAAG |  |
| *OsOr*_sg2 2nd-Fw | GTGGCGGGTCGCGTCCT |  |
| *OsOr*_sg2 2nd-Rv | GATTGACCCACGACCAACC |  |
| *OsOr*_sg3 1st-Fw | TTGGTGCTGGAGTACGACTG | NGS analysis for *OsOr*_sg2 |
| *OsOr*_sg3 1st-Rv | TGCTGATTACCAAGCAAGGA |  |
| *OsOr*_sg3 2nd-Fw | TATTGCACGGAGCTCATGTT |  |
| *OsOr*_sg3 2nd-Rv | CCAAGCTTACACTATTGCCATTT |  |
| *OsOr*_sg1 cDNA 1st-Fw | CATTGAAGGGCCTGAGACAG | NGS analysis for *OsOr*_sg1  in plants RNA |
| *OsOr*_sg1 cDNA 1st-Rv | GGTACTTGAACAGCGAGCAC |  |
| *OsOr*_sg1 cDNA 2nd-Fw | TCGCAGGTTGAGAATACAACA |  |
| *OsOr*_sg1 cDNA 2nd-Rv | CCTCCCACACCGAGTTTAAG |  |
| *OsPSY1*_RT-qPCR_Fw | GTCTGGGCGTCTCTGTTGTT | Expression analysis of *OsPSY1* |
| *OsPSY1*_RT-qPCR_Rv | CGCCCTCTTTGTGAAGTTGT |  |
| *OsPSY2*_RT-qPCR_Fw | TGATGCTATCGAAGCAAACG | Expression analysis of *OsPSY2* |
| *OsPSY2*_RT-qPCR_Rv | GCATAAGCGACCGGTAAAGA |  |
| *OsPSY3*_RT-qPCR_Fw | GAGATCGAGGCCAACGATTA | Expression analysis of *OsPSY3* |
| *OsPSY3*_RT-qPCR_Rv | GGGGAGCATGAGTGATCTGT |  |
| *OsPDS*_RT-qPCR_Fw | GGTTGCAATGGAAGGAACAC | Expression analysis of *OsPDS* |
| *OsPDS*_RT-qPCR_Rv | CATTTAAGGGTGCAGGCAAT |  |
| *OsZDS*_RT-qPCR_Fw | GCGAGTCACCAGGAAATGAT | Expression analysis of *OsZDS* |
| *OsZDS*_RT-qPCR_Rv | CCCTTCCATGCTGTCAATGT |  |
| *OsCRTISO*_RT-qPCR_Fw | TGCCTGCTGATACTGATTGC | Expression analysis of *OsCRTISO* |
| *OsCRTISO*_RT-qPCR_Rv | ATGGGTCAAGCACTGTAGGG |  |
| *OsLCYe*_RT-qPCR_Fw | CTTGGTTCGACGCTTTCTTC | Expression analysis of *OsLCYe* |
| *OsLCYe*_RT-qPCR_Rv | GGTTCATTCGCATTTGGTTC |  |
| *OsLCYb*_RT-qPCR_Fw | TGGACATCCTCCTCAAGCTC | Expression analysis of *OsLCYb* |
| *OsLCYb*_RT-qPCR_Rv | AAGAAGAGCCTCGACGACAG |  |
| *OsCCD1*_RT-qPCR_Fw | TTCTTTGTCCACGACGAGAAT | Expression analysis of *OsCCD1* |
| *OsCCD1*_RT-qPCR_Rv | AACTCGGCTTGGTAGCTCAA |  |
| *OsCCD4a*_RT-qPCR_Fw | GCTTCAACATCATGCACTCG | Expression analysis of *OsCCD4a* |
| *OsCCD4a*_RT-qPCR_Rv | GCTCGATGGAGAGGACGTT |  |
| *OsCCD4b*_RT-qPCR_Fw | ACTTTGATCTCGTCGGCTCT | Expression analysis of *OsCCD4b* |
| *OsCCD4b*_RT-qPCR_Rv | CGTCGATGTTGTCTGGAACA |  |
| *OsActin*_RT-qPCR_Fw | ATGGTTGGGATGGGTCAAAAA | Expression analysis of *OsActin* |
| *OsActin*_RT-qPCR_Rv | TCTTTAATGTCACGGACGATT |  |

**Supplementary Table S2.** Mutation frequencies at on-target and potential off-target sites among selected sgRNA targets of the *OsOr* gene in rice genome using CRISPR RGEN tools (http://www.rgenome.net/cas-designer/). PAM is presented in red, and the selected sgRNA is underlined.

| sgRNA No. | RGEN Target (5' to 3') | Direction | GC content  (%, w/o PAM) | Out-of frame score | Mismatches | | | |
| --- | --- | --- | --- | --- | --- | --- | --- | --- |
|  |  |  |  |  | 0 | 1 | 2 | 3 |
| *Osor_sg1* | GAAGTATATATACTTACCAGGGG | - | 30.0 | 71.9 | 1 | 0 | 0 | 0 |
| *Osor_sg2* | ATGCTGGCCTGCAGCGGCCTCGG | + | 70.0 | 61.3 | 1 | 0 | 0 | 0 |
|  | ACGCCTGCAGCAGCGTCGTTCGG | + | 65.0 | 71.2 | 1 | 0 | 0 | 0 |
|  | GCAGCAGCGTCGTTCGGCTCTGG | + | 70.0 | 60.8 | 1 | 0 | 0 | 0 |
| *Osor_sg3* | AGTCCAGGATTTTGAAAAGCTGG | + | 40.0 | 23.3 | 1 | 0 | 0 | 0 |
|  | GCTGGACTTGCAGGAGATTCAGG | + | 55.0 | 56.6 | 1 | 0 | 0 | 0 |
|  | AGGAGATTCAGGATAATATTAGG | + | 30.0 | 72.6 | 1 | 0 | 0 | 0 |

**Supplementary Table S3**. Mutation percentage and types of targeted mutagenesis of *OsOr* gene using the CRISPR-Cas9 system.

| Target region | No. of plants examined | No. of plants transformed | No. of plants with mutations | Putative homozygous mutations | | Putative  bi-allelic mutations | | Putative heterozygous mutations | |
| --- | --- | --- | --- | --- | --- | --- | --- | --- | --- |
|  |  |  |  | Number | % | Number | % | Number | % |
| *Osor*_sg1 | 20 | 19 | 18 (94.7%) | 4 | 22.2 | 12 | 66.7 | 2 | 11.1 |
| *Osor*_sg2 | 20 | 20 | 18 (90.0%) | 2 | 11.1 | 5 | 27.8 | 11 | 61.1 |
| *Osor*_sg3 | 16 | 16 | 14 (87.5%) | 2 | 14.3 | 9 | 64.3 | 3 | 21.4 |

**Supplementary Table S4.** Analysis of carotenoid content. Carotenoids were extracted from the WT, OC#1, OC#3, and OC#17 lines using a 0.01% solution of butylated hydroxytoluene in acetone. and analyzed using an Agilent 1260 high-performance liquid chromatography (HPLC) system.

| *Line* | Lutein | | 13Z-*β*-carotene | | *α*-carotene | | *β*-carotene | | 9Z-*β*-carotene | | others | | total | |
| --- | --- | --- | --- | --- | --- | --- | --- | --- | --- | --- | --- | --- | --- | --- |
|  | Mean | SD | Mean | SD | Mean | SD | Mean | SD | Mean | SD | Mean | SD | Mean | SD |
| OC #17 | 4.9 | 0.6 | 7.81 | 0.14 | 35.4 | 2.4 | 51.50 | 7.25 | 4.09 | 0.28 | 42.38 | 8.04 | 146.16 | 18.70 |
| OC #3 | 4.7 | 0.2 | 1.88 | 0.11 | 13.8 | 2.0 | 16.91 | 2.34 | 2.34 | 0.29 | 31.62 | 1.80 | 71.19 | 6.81 |
| OC #1 | 5.5 | 0.4 | 0.85 | 0.14 | 3.17 | 0.23 | 4.67 | 0.68 | 0.91 | 0.08 | 26.81 | 2.78 | 41.94 | 4.29 |
| WT | 2.3 | 0.2 | 0.29 | 0.04 | 0.67 | 0.11 | 0.77 | 0.04 | 0.42 | 0.08 | 10.98 | 1.73 | 15.48 | 2.23 |

*Data are expressed as mean (the average value of content for dry weight) and SD (the standard deviation value) of three independent experiments. Carotenoid contents were calculated as µg g^-1^ dry weight of callus tissues.


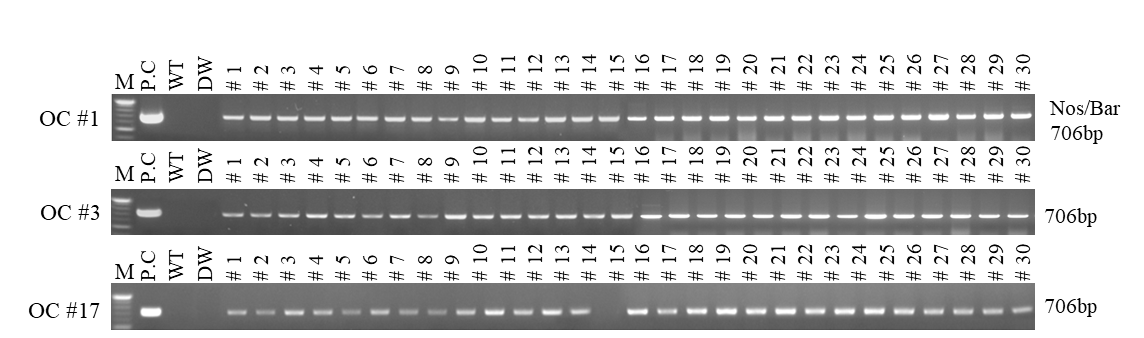


**Supplementary Figure S1.** PCR analysis using bar-gene-specific primers to confirm the transgenic callus lines. M, molecular marker; PC, positive control; WT, wild-type; DW, water.


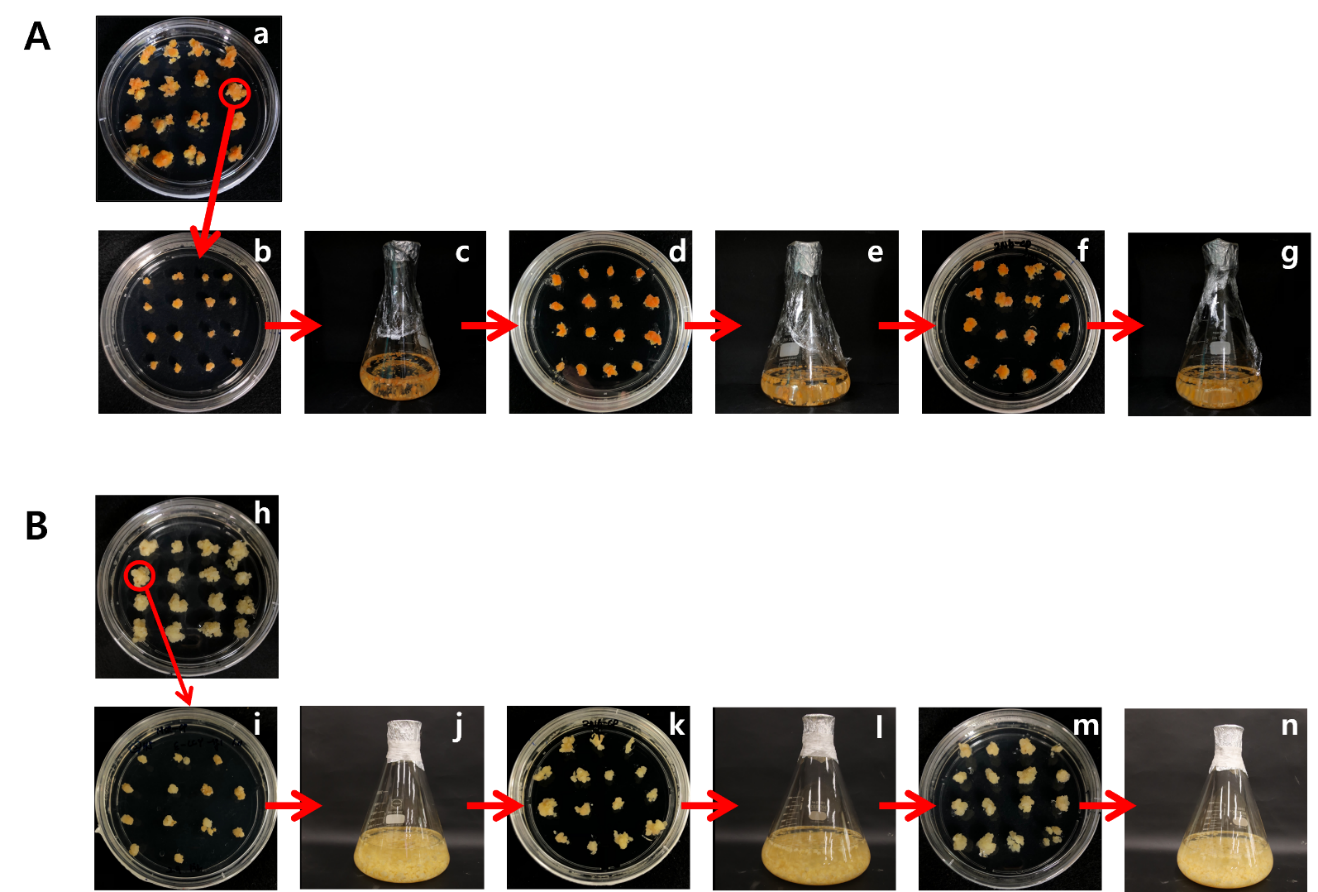


**Supplementary Figure S2.** Photographs of the subculture process for the generation of a single callus line. The OC#1, OC#3, and OC#17 lines were selected by targeted mutagenesis using the CRISPR-Cas9 system. These lines were subjected to several subcultures for a single cell line and maintained in AA medium.
