# Supplementary figures and images for: Biochemical Characterization of Orange-Colored Rice Calli Induced by Target Mutagenesis of OsOr Gene
Source: Plants (Basel). 2022 Dec 22;12(1):56. doi: 10.3390/plants12010056 (PMC9823629; doi:10.3390/plants12010056)

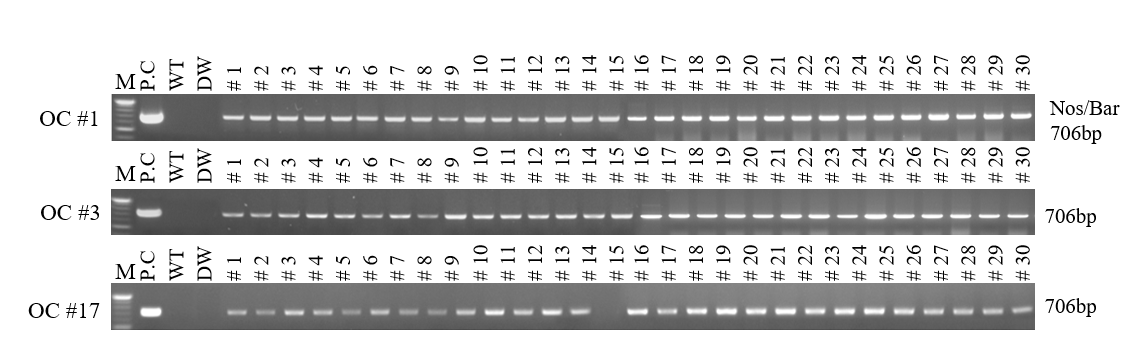

Supplement: Supplementary file 1 [file plants-12-00056-s001.zip › supple. Fig S1.tif]

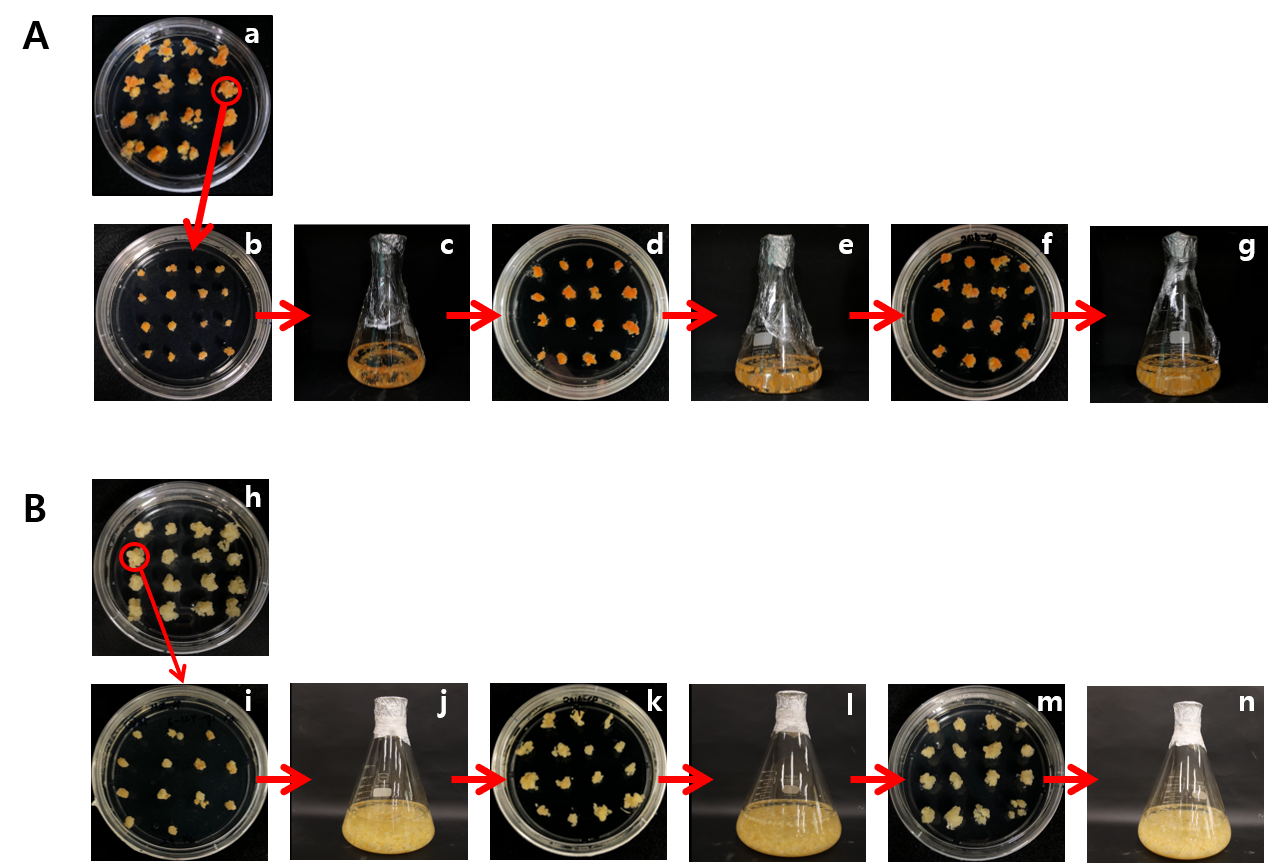

Supplement: Supplementary file 1 [file plants-12-00056-s001.zip › supple. Fig S2.tif]
